# Is day 7 culture necessary for in vitro fertilization of cryopreserved/warmed human oocytes?

**DOI:** 10.1186/s12958-020-0565-9

**Published:** 2020-01-18

**Authors:** Xiangli Niu, Cassie T. Wang, Richard Li, Ghassan Haddad, Weihua Wang

**Affiliations:** 1 Research Center for Reproductive Medicine, Reproductive Hospital of Guangxi Zhuang Autonomous Region, Nanning, Guangxi China; 2 Prelude-Houston Fertility Laboratory, Houston Fertility Institute, 2500 Fondren Rd., Suite 350, Houston, TX 77063 USA; 3 Houston Fertility Institute, 2500 Fondren Rd., Suite 350, Houston, TX 77063 USA

**Keywords:** Oocytes, Warming, Blastocysts, Day 7 culture, Aneuploidy

## Abstract

**Background:**

Human embryos are usually cultured to blastocyst stage by Day 5 or 6 after insemination. However, some embryos grow slowly and reach blastocyst stage at Day 7. Acceptable live birth rates have been reported after transfer of Day 7 blastocysts resulted from fresh oocyte in vitro fertilization (IVF). It is unknown whether an extended embryo culture to Day 7 is necessary for cryopreserved oocyte IVF to obtain more transferrable blastocysts.

**Methods:**

In this study, 455 oocytes from 57 cycles were warmed, inseminated, and the resulting embryos were cultured by Day 7 to examine blastocyst development after extended culture. Fifty one blastocysts from 16 cycles were biopsied to examine embryo aneuploidies.

**Results:**

It was found that 35.1% of the cycles had Day 7 blastocysts, and 3.5% of the cycles had only Day 7 blastocysts. Day 7 blastocysts accounted for 15.6% of total blastocysts. The proportion of top quality of blastocysts was lower at Day 7 than at Day 5 or 6. However, no differences were observed on aneuploid blastocyst rates among Days 5, 6 and 7. Similar clinical pregnancy, ongoing pregnancy and embryo implantation rates were obtained after Day 7 blastocyst transfer as compared with Day 5 or 6 blastocyst transfer.

**Conclusion:**

These results indicate that embryos from oocyte warming cycles should be cultured to Day 7 if they do not reach to blastocyst stage by Day 6 so that number of usable blastocysts can be increased.

## Background

Oocyte cryopreservation has become one of the most important human assisted reproductive technologies, which provides approaches of fertility preservation for women who want to delay childbearing [1], and for young cancer patients before treatment [2, 3]. It also facilitates the establishment of donor egg bank [4].

The procedures for in vitro fertilization (IVF) and embryo culture did not have any difference between cryopreserved/warmed oocytes and fresh oocytes. However, recently, it has been found by time-lapsing analysis and morphokinetic that cryopreserved/warmed oocytes had about 1 h of delayed fertilization and embryo development [5]. Delayed embryo development is very often in human IVF even fresh oocytes are used, which is indicated by a delayed blastocyst formation. Day 7 blastocyst accounts for ~ 5–8% of total blastocysts [6]. Although most human IVF laboratories culture human embryos to Day 6, an extended Day 7 culture is necessary for some patients, especially if patients have limited number of oocytes [6–8]. It has been reported that Day 7 blastocysts had lower implantation potential as compared with Day 5/6 blastocysts, however, transfer of Day 7 blastocysts can lead to acceptable live birth rates [9, 10].

For cryopreserved oocytes from oocyte bank, oocyte number is very limited, usually 6–8 per cycle, thus the number of embryos developed to blastocyst stage is much fewer than that from most fresh oocyte IVF cycles. Therefore, it may be necessary to extend embryo culture to Day 7 so that slow growing embryos can reach to blastocyst stage. In our clinic, we started to Day 7 culture for all oocyte warming cycles from 2019, and our preliminary data showed that implementation of Day 7 culture for oocyte warming IVF cycles increased overall blastocyst number.

## Results and discussion

In this study, 455 cryopreserved donor oocytes were warmed for 57 recipient cycles. Warmed oocytes were inseminated by intracytoplasmic sperm injection and all embryos were extended to Day 7 culture if they did not reach blastocysts by Day 6. As shown in Table 1, the overall survival, fertilization, cleavage and blastocyst rates were 94.1, 77.3, 94.9 and 56.2%, respectively. Days 5, 6 and 7 blastocysts were 34.3, 50.0 and 15.6%, respectively. The proportion of blastocysts at Day 7 appears to be higher than that with fresh oocyte IVF (< 5%) reported previously [6]. We found that 20 cycles (35.1%) had Day 7 blastocysts, which is almost doubled as compared with fresh oocyte IVF (18.5%) [9]. These results may indicate that embryo development of cryopreserved oocytes is slower in some cases although we still do not know what caused the slow embryo development. We also found that 3.5% of the oocyte warming cycles had only Day 7 blastocysts, which was the same as that with fresh oocyte IVF [9].

**Table 1:** Laboratory outcomes after cryopreserved/warmed oocyte IVF and extended embryo culture to Day 7

No. of warming cycle	57
No. of oocytes warmed	455
Mean ages of recipients	42.8 ± 6.3
Mean No. of oocytes warmed	7.98 ± 1.87
No. of oocytes survived	428 (94.1%)
No. of oocytes fertilized	331 (77.3%)
No. of oocytes cleaved	314 (94.9%)
Total No. of blastocysts	186 (56.2%)
At Day 5	64 (34.4%)
At Day 6	93 (50.0%)
At Day 7	29 (15.6%)
No. of cycles with Day 7 blastocysts	20 (35.1%)
No. of cycles with only Day 7 blastocysts	2 (3.5%)

Day 7 blastocysts had poorer quality as compared with Day 5 or Day 6 blastocysts [9, 11, 12]. The similar results were also observed in the present study. We found that only 20.7% of Day 7 blastocysts were top quality of blastocysts with both good inner cell mass (ICM) and good trophectoderm (TE), which was significantly lower than those at Day 5 (73.4%) or Day 6 (57.0%). However, we found that 41.4% of Day 7 blastocysts still have either a good ICM or a good TE. Although it is not very clear which (ICM or TE) is more important for a blastocyst to become a live birth, a good ICM [13], or good TE [14] is necessary for a blastocyst implantation.

Although previous studies found that more Day 7 blastocysts were aneuploid as compared with Day 5 or Day 6 blastocysts, suggesting that slow growing embryos may be resulted from oocyte aneuploidy [6, 9, 11, 12]. In the present study, 16 cycles had blastocyst biopsy for preimplantation genetic testing for aneuploidies (PGT-A), and a total of 51 blastocysts (13 at Day 5, 31 at Day 6 and 7 at Day 7) were biopsied. The euploid blastocyst rates were 53.9, 61.3 and 57.1%, respectively, with no statistical difference being observed among Days 5, 6 and 7 blastocysts. These results may indicate that oocytes quality is different from these studies. In the present study, donor oocytes were used while autologous oocytes at various ages were used in the previous studies [9, 11, 12]. Therefore, slow growing embryos from cryopreserved donor oocytes may not be due to oocyte quality, but due to the procedures of oocyte cryopreservation and warming, which results in delayed embryo development in some cases.

In the present study, there were 35 fresh blastocyst transfers and 16 frozen embryo transfer (FET). There were no statistical differences between fresh blastocyst transfer and FET in terms of clinical pregnancy rates (60.0 vs 56.3%), ongoing pregnancy rates (54.3 vs 50.0%) and embryo implantation rates (53.9 vs 58.8%).

Acceptable live birth rates have been obtained after Day 7 blastocyst transfers [6–10]. However, all of the data in these previous reports were from autologous and fresh donor oocyte IVF. As shown in Table 2, in the present study with frozen donor eggs, we did not find the difference among Days 5, 6 and 7 blastocyst transfer in terms of positive β-hCG rate, clinical pregnancy rate, ongoing pregnancy rate and implantation rate between days 5, 6 and 7 blastocyst transfer. Three patients had Day 7 blastocyst transfer (one with a euploid blastocyst after PGT-A, and two with no PGT-A tested blastocysts), all three were clinical pregnant and 2 are ongoing pregnant. These results indicate that pregnancy rate is also acceptable after transfer of Day 7 blastocysts resulting from frozen/warmed eggs.

**Table 2:** Comparison of clinical outcomes of Day 5, 6, and 7 blastocyst transfers

Transfer categories	No. of ET	Positive β-hCG (%)	Clinical pregnancy (%)	Ongoing pregnancy (%)	No. of embryos transferred	Implanted/transferred embryos (%)
Day 5 blast	39	26 (66.7%)	24(61.5%)	22 (56.4%)	1.10 ± 0.31	24/43 (55.8%)
Day 6 blast	9	4 (44.4%)	3 (33.3%)	3 (33.3%)	1.0 ± 0.0	3/9 (33.3%)
Day 7 blast	3	3 (100%)	3 (100%)	2 (66.7%)	1.33 ± 0.58	4/4 (100%)
*P* value^a^	NA	0.4	0.23	0.41	0.60	0.18

^a^No Statistical differences were observed among Days 5, 6 and 7 blastocysts in terms of β-hCG, clinical pregnancy, ongoing pregnancy rates, mean number of embryos transferred and embryo implantation rates

For fresh oocyte donation, recipients usually receive many oocytes and high quality of oocytes usually produces enough blastocysts at Days 5 and 6, thus very few laboratory may culture embryos from donor oocytes to Day 7. This may be the reason that no data is published on the transfer of Day 7 blastocysts resulted from donor oocytes. However, most IVF clinics now switch fresh oocyte to frozen oocyte IVF when donated oocytes are used [15], and 6 oocytes are warmed for each cycle in most cases. This change may require the implementation of extended embryo culture to Day 7 so that more usable/transferrable blastocysts can be obtained for patients.

## Conclusions

In conclusion, our preliminary data with limited case numbers indicate that Day 7 culture is necessary for cryopreserved/warmed oocyte IVF cycles, especially for those cycles with limited number of oocytes being warmed. The proportions of cycles with Day 7 blastocysts and proportions of blastocysts obtained at Day 7 appear to be higher than those observed with fresh oocyte IVF. In addition, our limited data indicate that aneuploid rate with frozen donor oocytes did not differ between Day 7 blastocysts and Days 5/6 blastocysts although quality of Day 7 blastocysts is not as good as quality of Day 5/6 blastocysts. Transfer of Day 7 blastocysts resulted in successful embryo implantation and clinical pregnancy, and both embryo implantation rate and clinical pregnant rate were not different from those with Day 5 or Day 6 blastocyst transfer. Putting together, these data suggest that the implementation of Day 7 culture for those embryos that have not developed to blastocysts by Day 6 is necessary in oocyte warming cycles.

## Data Availability

The primary data for this study is available from corresponding author on reasonable request.

## Abbreviations

FET: Frozen embryo transfer
ICM: Inner cell mass
IVF: In vitro fertilization
PGT-A: Preimplantation genetic testing for aneuploidies
TE: Trophectoderm
